# Elexacaftor/Tezacaftor/Ivacaftor Effect on Bone Density and Body Composition: A Retrospective Analysis

**DOI:** 10.1002/ppul.71280

**Published:** 2025-09-09

**Authors:** Susanne Ursula Trost, Tasma Harindhanavudhi, Qi Wang, Anvitha Ankireddypalli, Azmi Simrah, Sreekant Avula, Amir Moheet

**Affiliations:** ^1^ Department of Medicine, Division of Diabetes, Endocrinology and Metabolism University of Minnesota Minneapolis Minnesota USA; ^2^ Clinical and Transitional Science Institute University of Minnesota Minneapolis Minnesota USA

**Keywords:** body composition, bone density, bone health, cystic fibrosis, elexacaftor/tezacaftor/ivacaftor, fat mass

## Abstract

**Background:**

The approval of cystic fibrosis transmembrane conductance regulator modulators elexacaftor/tezacaftor/ivacaftor (ETI), has significantly improved pulmonary function for people with cystic fibrosis (pwCF). However, the effects on CF‐related bone disease and body composition remain unclear.

**Methods:**

This retrospective real‐world study examined adults with CF who received ETI treatment. Bone density and body composition were measured via dual‐energy X‐ray absorptiometry (DXA) 1.8 (SD 0.7) years before (preDXA) and 1.5 (SD 0.4) years after ETI initiation (postDXA1). In a subgroup, measurements were also available 4 (SD 0.6) years after initiation (postDXA2).

**Results:**

The study included 74 pwCF, of whom 42% were female, with an average age of 38.9 (SD 9.3) years at ETI initiation. Bone density decreased significantly at the spine (*p* = 0.02), left hip (*p* < 0.01), and right hip (*p* < 0.01) from pre‐ to postDXA1. No significant change in bone density was observed when comparing postDXA1 to postDXA2. Body composition analysis revealed significant increases in weight in both females (*p* < 0.01) and males (*p* < 0.01), as well as increases in fat mass in females (*p* < 0.01) and males (*p* < 0.01), without significant changes in lean mass (*p* = 0.4).

**Conclusions:**

Bone density declined significantly during the study period, with the most pronounced decline occurring in the first 1.5 years after ETI initiation. However, this decline appeared to stabilize in participants with longer follow‐up at 4 years post‐ETI treatment, suggesting potential bone‐protective effects with prolonged therapy. Body weight and fat mass increased early after ETI initiation, suggesting metabolic changes accompanying CFTR modulation.

## 1 Introduction

Cystic fibrosis (CF) is a progressive, multi‐system genetic disorder caused by mutations in the cystic fibrosis transmembrane conductance regulator (CFTR) gene [1]. Life expectancy for people with CF (pwCF) has increased dramatically from 10 years of age in 1962 to 53 years for pwCF born in 2021 [2]. The development of CFTR modulators has revolutionized CF care, offering targeted therapies that address the underlying protein dysfunction rather than merely treating symptoms [3]. Elexacaftor/tezacaftor/ivacaftor (ETI; Trikafta), approved in October 2019 for pwCF with at least one copy of the F508del mutation and later expanded to 177 additional ETI‐responsive mutations in December 2020, represents the most significant therapeutic advancement in CF treatment to date, with 85.5% of pwCF in the United States now eligible for this therapy [3].

ETI has demonstrated remarkable efficacy in improving pulmonary function, reducing respiratory symptoms, and enhancing nutritional status across diverse CF populations [4, 5]. Clinical trials have consistently shown substantial improvements in forced expiratory volume in 1 s (FEV1), reductions in pulmonary exacerbations, and improved quality of life measures. However, as pwCF experiences longer lifespans with these highly effective therapies, non‐pulmonary complications of CF have gained increased clinical relevance [6, 7].

CF‐related bone disease (CFBD) represents a significant comorbidity affecting a substantial proportion of adults with CF [6]. CFBD was first noted to be associated with low bone density in the forearm in the literature in 1979 [8]. In 1998, Aris et al found an approximately twofold increase in reported fractures in adulthood and additionally noted a significant number of unreported vertebral and rib fractures [9]. A study in 2022 showed that 17.8% of adults with CF had a history of fracture [10]. The pathophysiology of CFBD is multifactorial, involving chronic pulmonary inflammation [11], malabsorption of calcium and fat‐soluble vitamins (particularly vitamins D and K), malnutrition, low body weight, glucocorticoid treatment, and direct effects of CFTR dysfunction on bone‐forming osteoblasts [6].

Similarly, body composition abnormalities have long been recognized as important determinants of clinical outcomes in CF [12, 13]. Traditional CF care has focused on preventing malnutrition and promoting weight gain, as higher body mass index (BMI) correlates with better pulmonary function and survival. However, the relationship between body composition and health outcomes in the era of highly effective modulator therapy requires re‐evaluation, particularly as concerns about overweight and obesity in CF populations emerge [14, 15].

The effects of CFTR modulators on bone health and body composition remain incompletely understood. Early studies with ivacaftor showed mixed results regarding bone density changes, with some reporting improvements [16] and others showing no change in bone density after 1−2 years of treatment [17, 18]. Data from the British and American cystic fibrosis registries by Bessonova et al. reported broadly decreased bone and joint complications with ivacaftor treatment; however, they did not include bone density data [19]. Limited data exist specifically for ETI's impact on bone health, with only small pilot studies providing preliminary insights. Gur et al. demonstrated increased bone density at the hip and spine in nine pwCF after 3 months of ETI treatment [20]. Similarly, CFTR modulators consistently promote weight gain [17, 21, 22, 23, 24, 25, 26, 27, 28, 29]; the composition of this weight gain—particularly the relative contributions of fat mass versus lean mass—has important implications for long‐term health outcomes. Mixed results are seen in studies, including weight increase primarily comprise fatmass with no or only a small increase in lean mass [22, 23, 24, 25, 26], while other studies report an increase in fat mass as well as lean mass [27, 28, 29]. This study addresses these knowledge gaps by providing a comprehensive analysis of bone density and body composition changes in adults with CF following ETI initiation. Using dual‐energy X‐ray absorptiometry (DXA) scanning data from a real‐world clinical setting, we examined both short‐term (1.5 years) and longer‐term (4 years) effects of ETI treatment on these important non‐pulmonary outcomes.

## 2 Methods

### 2.1 Population

This retrospective longitudinal study included adults aged ≥ 18 years followed at the Minnesota CF Center, University of Minnesota from January 1, 2018, until March 15, 2025, who were part of the CF Database. Inclusion criteria were ETI treatment and bone density (DXA) scans both before and after ETI initiation. Exclusion criteria were status post lung transplant, pregnancy, and current or prior treatment with antiresorptive or anabolic agents for bone health.

### 2.2 Bone Density

Bone density screening is recommended in the “Guide to Bone Health and Disease in Cystic Fibrosis” [6]. The Minnesota CF Center aims to screen bone density every 2 years in all adults with CF. All DXA scans were performed at M Health Fairview Imaging at the medical center using a GE Lunar Prodigy DF + 15769 scanner. All follow‐up scans were performed on the same machine.

Data from the DXA scans were collected at four time points relative to ETI initiation: (1) Remote DXA—the scan preceding the pre‐ETI scan, obtained on average 3.8 (SD 1.9) years before ETI initiation and available for 57 (77%) participants; (2) pre‐DXA—the most recent scan before ETI initiation, obtained on average 1.8 (SD 0.7) years before treatment start; (3) postDXA1—the first scan following ETI initiation, obtained on average 1.5 (SD 0.4) years after treatment start; and (4) postDXA2—the second posttreatment scan, obtained on average 4.0 (SD 0.6) years after ETI initiation and available for 51 (69%) participants. Bone mineral density with *Z*‐scores at the lumbar spine, total left hip, and total right hip were documented from available bone density reports. *Z*‐scores were used to follow bone density in all patients over time.

### 2.3 Body Composition

Body composition data were acquired simultaneously with bone density measurements. According to manufacturer specifications, pwCF were positioned appropriately in the supine position. Data from whole body fat mass and lean mass were considered for body composition analysis.

### 2.4 Pulmonary Function Test

FEV1 measurements from routine CF clinic visits closest to the pre‐ and post‐DXA timepoints were analyzed.

### 2.5 Statistical Analyses

Descriptive statistics were calculated and presented using means and standard deviations for continuous variables and frequencies and percentages for categorical variables. Linear mixed models adjusted for gender were used to evaluate changes over time (remote, pre, post1, and post2). Models included a fixed effect of time and a random intercept to account for correlations among repeated measures within subjects. If the overall *F* test for fixed effect of time was significant, pairwise comparisons (remote vs. pre, pre vs. post1, and post1 vs. post2) were conducted with Tukey adjustment for multiple comparisons. Statistical analyses were performed in SAS version 9.4 (SAS Institute Inc., Cary, NC). *p* values of less than 0.05 were considered statistically significant.

### 2.6 Institutional Review Board (IRB) Approval

The study design was approved by the University of Minnesota Institutional Review Board. Waiver of consent was requested for this chart review study, as participants in the CF database had already consented for their data to be used for future research.

## 3 Results

### 3.1 Subject Characteristics

Of the 74 pwCF (Table 1), 31 were female. Average age at ETI treatment initiation was 38.9 years (SD 9.3). All pwCF started ETI treatment between November 2019 and December 2020. Forty‐nine (66%) had been on prior modulator treatment; all except two pwCF had at least one F508del mutation. BMI before treatment start was 24.3 kg/m^2^ (SD 2.9). The vast majority of patients were treated with pancreatic enzymes (69, 93%), and 34 (46%) had cystic fibrosis‐related diabetes (CFRD).

**Table 1:** Baseline characteristics of pwCF at time of initiation of elexacaftor/tezacaftor/ivacaftor (ETI).

	*n* = 74
Gender, female, *n* (%)	31 (42.0%)
Age (years) at ETI start, mean (SD)	38.9 (9.3)
CFRD at baseline, *n* (%)	34 (45.9%)
Prior modulator, *n* (%)	49 (66.2%)
Chronic steroid treatment, *n* (%)	4 (5.4%)
Pancreatic insufficiency, *n* (%)	69 (93.2%)
Creatinine (mg/dL), mean (SD)	0.84 (0.17)
25‐Hydroxy vitamin D (ng/mL), mean (SD)	38.7 (14.4)

Abbreviations: CFRD, cystic fibrosis‐related diabetes; ETI, elexacaftor/tezacaftor/ivacaftor; pwCF, people with cystic fibrosis.

### 3.2 Bone Density

Bone density from all pwCF were obtained on average 1.8 (SD 0.7) years before ETI (preDXA) and 1.5 (SD 0.4) years post‐ETI (postDXA1) initiation. Remote bone density was obtained 3.8 (SD 1.9) years before ETI initiation and was available for 57 (77%) pwCF (remoteDXA). Fifty‐one (69%) pwCF had a second bone density scan 4.0 (SD 0.6) years post‐ETI initiation (postDXA2).

**Figure 1:**
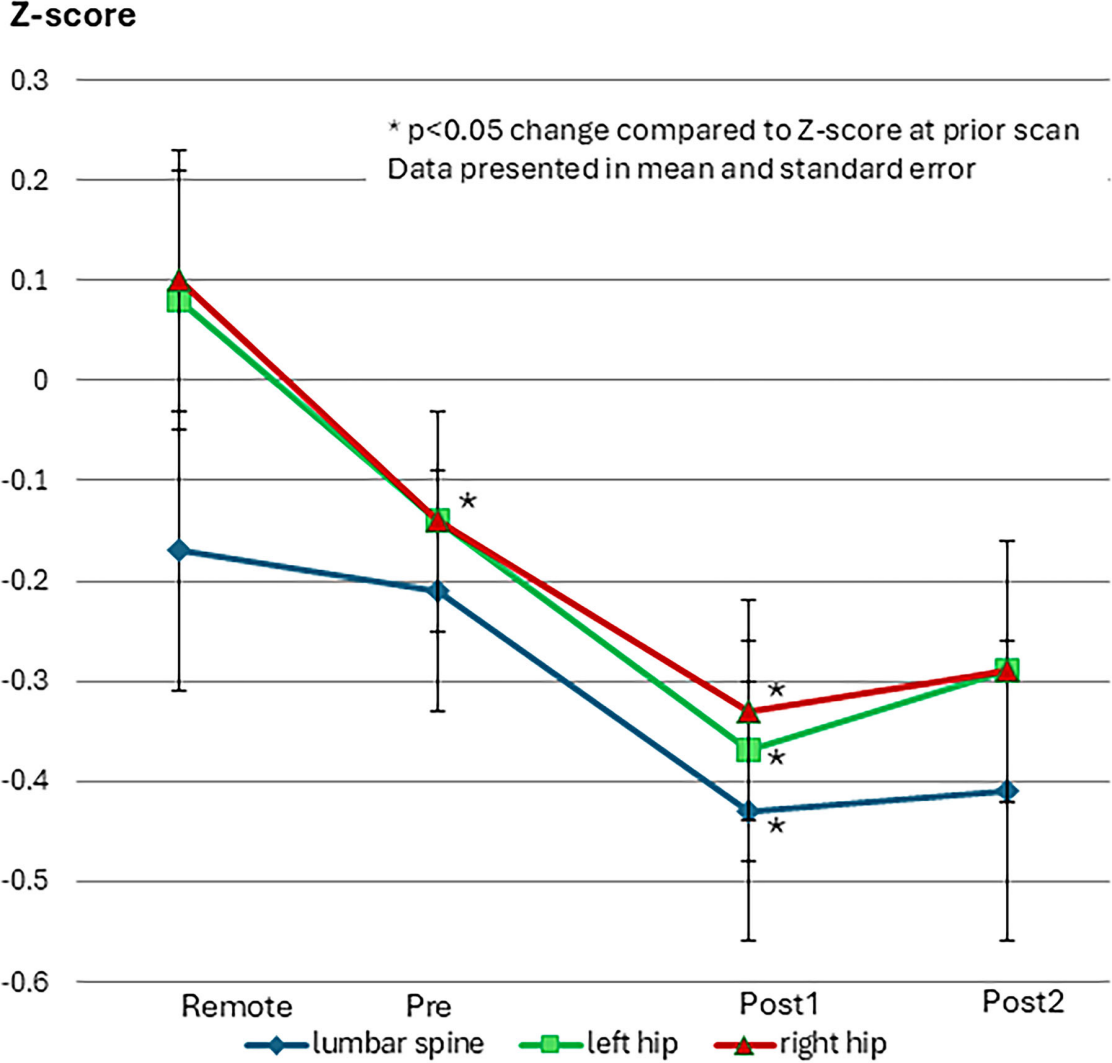
Bone density before and after elexacaftor/tezacaftor/ivacaftor (ETI) initiation: *Z*‐scores at the left and right total hip and lumbar spine. Remote: the DXA scan preceding the most recent DXA done before ETI start. Pre: the most recent DXA before ETI start. Post1: the first DXA scan following the initiation of ETI. Post2: the second DXA scan after the initiation of ETI. [Color figure can be viewed at wileyonlinelibrary.com]

Before ETI initiation at the PreDXA, average bone density showed *Z*‐scores of −0.21, −0.14, and −0.14 at the lumbar spine, left total hip, and right total hip, respectively (Table 2). Bone density decreased significantly during the entire study from remote DXA to postDXA2 at all sites. This decrease continued in the 2.8 years from pre‐ETI treatment (preDXA) to posttreatment (postDXA1) at all sites, including lumbar spine (*p* < 0.01), and left and right total hip (both *p* < 0.01, Table 2, Figure 1).

**Table 2:** Bone mineral density based on DXA scan in pwCF before and after ETI initiation.

Bone mineral density						Pairwise comparison *p* value		
	Remote (*n* = 57)	Pre (*n* = 74)	Post1 (*n* = 74)	Post2 (*n* = 51)	Overall *p* value	Remote versus pre	Pre versus post1	Post1 versus post2
*Z*‐score lumbar spine	−0.17 (1.03)	−0.21 (1.01)	−0.43 (1.12)	−0.41 (1.09)	< 0.01	0.69	< 0.01	0.98
*Z*‐score left hip	0.08 (0.97)	−0.14 (0.96)	−0.37 (0.96)	−0.29 (0.95)	< 0.01	0.07	< 0.01	1.00
*Z*‐score right hip	0.10 (1.00)	−0.14 (0.92)	−0.33 (0.97)	−0.29 (0.95)	< 0.01	< 0.01	< 0.01	0.99

*Note:* Remote: the DXA scan preceding the most recent DXA done before ETI start.

Pre: the most recent DXA before ETI start.

Post1: the first DXA scan following the initiation of ETI.

Post2: the second DXA scan after the initiation of ETI.

Abbreviations: DXA, dual energy X‐ray absorptiometry; ETI, elexacaftor/tezacaftor/ivacaftor; pwCF, people with cystic fibrosis.

The magnitude of bone density decline observed was clinically meaningful, with *Z*‐score decreases ranging from 0.19 to 0.23 standard deviations between pre‐ETI and postDXA1 measurements across all sites. These changes represent a shift toward lower bone density relative to age‐matched peers, which is concerning given that many participants had baseline *Z*‐scores already below the population mean.

However, the second DXA obtained an average of 4.0 (SD 0.6) years after ETI start (postDXA2) in a subgroup of 51 (68%) pwCF did not show significant change when compared to the first bone density after ETI start (postDXA1) at any of these sites (spine *p* = 0.99, left femur *p* = 1.00, right femur *p* = 0.99, Table 2, Figure 1). Analysis of DXA scans in 43 patients who had scans at all four time points showed a similar pattern of decreased bone density measured in the first post‐treatment scan and attenuation of decline at the second post‐treatment scan (see Table S1).

### 3.3 Body Composition

During the study, body weight, BMI, and fat mass increased significantly (*p *< 0.01) (Table 3), with no change in lean mass. In females, changes in body composition pre‐ and post‐DXA showed increases in total body weight from 61.7 (SD 10.3) to 64.7 (SD 9.7) kg (*p* = 0.01), increases in BMI from 23.7 (SD 3.3) to 24.8 (SD 2.9) kg/m^2^ (*p* = 0.03), and increases in fat mass from 21.6 (SD 7.6) to 25.0 (SD 7.4) kg (*p* < 0.01) (Table 3).

**Table 3:** Body composition per DXA scan in pwCF before and after ETI initiation.

Body composition						Pairwise comparison		
	Remote	Pre	Post1	Post2	Overall *p* value	Remote versus pre	Pre versys post1	Post1 versus post2
Female (*n* = 31)	*n* = 21	*n* = 31	*n* = 30	*n* = 21		*p* value		
Weight (kg)	58.6 (10.2)	61.7 (10.3)	64.7 (9.7)	66.1 (10.1)	< 0.01	0.29	0.01	0.78
BMI (kg/m^2^)	22.7 (3.5)	23.7 (3.3)	24.8 (2.9)	25.02.9)	< 0.01	0.22	0.03	0.99
Fat mass (kg)	19.3 (7.5)	21.6 (7.6)	25.0 (7.4)	25.1 (8.4)	< 0.01	0.54	< 0.01	0.99
Lean mass (kg)	39.9 (3.8)	40.1 (5.1)	39.9 (5.5)	41.0 (5.2)	0.50			
Male (*n* = 43)	*n* = 27	*n* = 33	*n* = 37	*n* = 24	Overall *p* value	*p* value		
Weight (kg)	77.9 (13.1)	76.8 (12.7)	80.5 (13.1)	80.9 (13.7)	< 0.01	0.91	< 0.01	0.96
BMI (kg/m^2^)	24.2 (3.2)	24.1 (3.1)	25.4 (3.4)	25.4 (3.4)	< 0.01	0.95	< 0.01	0.88
Fat mass (kg)	20.5 (8.3)	20.0 (8.5)	22.0 (9.3)	23.7 (9.1)	< 0.01	0.87	0.001	0.99
Lean mass (kg)	59.6 (5.9)	58.1 (5.7)	56.6 (10.6)	60.0 (5.9)	0.22			

*Note:* Remote: the DXA scan preceding the most recent DXA done before ETI start

Pre: the most recent DXA before ETI start.

Post1: the DXA scan following the initiation of ETI.

Post2: the second DXA scan after the initiation of ETI.

Abbreviations: DXA, dual energy X‐ray absorptiometry; ETI, elexacaftor/tezacaftor/ivacaftor; pwCF, people with cystic fibrosis.

Similarly, in men pre‐ and post‐ETI initiation, weight increased from 76.8 (SD 12.7) to 80.5 (SD 13.1) kg (*p* < 0.01), BMI from 24.1 (SD 3.2) to 25.4 (SD 3.4) kg/m^2^ (*p* < 0.01), and fat mass from 20.0 (SD 8.3) to 22.0 (SD 9.3) kg (*p* < 0.01) (Table 3).

### 3.4 Pulmonary Function

The pulmonary function measured as FEV1 at the time of the DXA before ETI initiation (preDXA) and after (postDXA1) showed an average increase of 10.3% from 2.62 (SD 0.37) to 2.90 (SD 0.03) L (*p* < 0.01).

### 3.5 Vitamin D

Vitamin D levels at the time of ETI initiation were available for 63 of 74 patients (Table 1). Among these, five pwCF had low 25‐hydroxy vitamin D [25(OH)D] levels (< 20 ng/mL), and five had insufficient levels (20−29 ng/mL). The remaining patients had documented vitamin D levels withing the normal range. A total of 71 out of 74 pwCF reported taking vitamin D supplements. Levels of 25(OH)D remained unchanged at 39.7 (SD5) ng/dL by the end of the study with nine pwCF still exhibiting inadequate vitamin D levels. No correlation was found between baseline vitamin D levels and bone mineral density at any of the DXA time points obtained.

## 4 Discussion

In this retrospective study of 74 pwCF, we analyzed the effects of ETI treatment on bone density and body composition in a real‐world setting. The decreased bone density was observed for the entire study with a significant decline in bone density from the ETI start to the average of 1.5 years of ETI treatment (postDXA1 vs. preDXA). However, no significant change in bone density was observed after prolonged ETI treatment for an average of 4 years (postDXA2 vs. postDXA1). The attenuation in the decline of bone density observed in participants with longer follow‐up suggests that ETI may have stabilizing effects on bone health over time. Similar results were found in the subgroup analysis of participants who had DXA data at all four time points.

A recent longitudinal study of 500 adults with CF showed normal bone density by age 25; however, a subsequent decline in bone density occurred earlier than in people without CF at ages younger than 45 [30]. A prior study by Gur et al. [20] showed increased bone density at the hip and spine in a group of nine pwCF after 3 months of ETI treatment. However, this study was small with a very short follow‐up duration. In comparison, our study had higher baseline bone density, a larger study size, and was conducted in a real‐world setting. The exclusion of pwCF undergoing lung transplants or those taking osteoporosis medications, as per our study design, explains why the average bone density in our study falls closely within the normal range.

Studies of ivacaftor's effects on bone density have shown mixed results, with some reporting improvements after short‐term treatment [16] while others found no significant changes after 1−2 years of therapy [17, 18]. These inconsistent findings may reflect differences in study populations, baseline bone health, and follow‐up duration, highlighting the need for longer‐term studies with larger cohorts such as the present analysis.

It appears that rapid improvement in CF treatment has led to improvements in nutrition, decreased inflammation, fewer CF exacerbations, and less glucocorticoid use [1], along with better vitamin D absorption [31], which should be beneficial for bone health. In this study, ETI treatment had no effect on vitamin D levels, which remained unchanged throughout. Additionally, no correlation was observed between baseline vitamin D levels and bone mineral density at any time point. CFBD continues to cause fractures with associated morbidities and loss of quality of life [10]. Furthermore, with the advent of highly potent CFTR modulator treatment, pwCF born today are expected to live into their 50s and older, which presents new challenges such as menopause with post‐menopausal bone loss and higher prevalence of peripheral vascular disease and peripheral neuropathy with longer‐standing diagnosis of CFRD [32], making CFRD an additional risk factor for fractures. It has also been noted that pwCF, even with ETI treatment, continue to carry a significant burden of respiratory inflammation, raising concerns for continued adverse impact on bone health in these pwCF [33].

Our study finds that weight gain associated with ETI treatment consists mostly of increased fat mass. Similar results were seen in prior studies [22, 23, 24, 25, 26]. A study evaluating body composition in 66 pwCF using opportunistic CT scan over a longer time period after ETI initiation reported mostly increases in fat mass tissue with only small gains in muscle mass [22]. However, as this study included measurements at only two time points post ETI initiation, it could not assess whether weight gain attenuates with longer treatment duration. The remainder of studies [23, 24, 25, 26] were small in size and duration of ETI treatment was relatively short, only 6−12 months. Studies showing ETI‐associated weight gain with a mix of fat and muscle increase were also small with about 1 year in duration, shorter than our study [27, 28]. In a longer study with Ivacaftor stabilization of weight after an initial 6‐month treatment period has been described [29]. Similarly, in this study, the subgroup of participants with a second scan posttreatment start showed no significant change in weight between PostDXA1 and PostDXA2 measurements, possibly indicating weight stabilization. The predominance of fat mass gain over lean mass gain has important clinical implications. While weight gain has traditionally been viewed as beneficial in CF, the composition of this weight gain may influence long‐term metabolic health, cardiovascular risk, and functional outcomes. The lack of lean mass increase suggests that ETI‐associated weight gain may not translate to improved muscle strength or physical function, highlighting the need for targeted interventions to promote lean body mass in this population [14, 15].

### 4.1 Strengths of This Study

This study provides data on non‐pulmonary outcomes in a moderate‐sized group of adults with CF on ETI treatment, including bone density and body composition in addition to clinical data and pulmonary function tests. The increase in FEV1 reported in the pivotal trials was in a similar range as in this study, supporting the validity of these real‐world data. Body composition was measured using DXA scanning, which is considered a reliable tool [34]. This differs from the bioelectrical impedance analyzers (BIA) used in other reports [22, 23, 24, 25, 27] and BIA with skin fold thickness [26] regarding changes in body composition with CFTR modulators. Our study was longer than in many prior studies, which is beneficial for a treatment intended to be long‐term, and the study size is significantly larger.

### 4.2 Limitations

Several limitations should be acknowledged. Body composition data availability varied across timepoints (61%−90% of participants), limiting our ability to detect changes in lean mass and potentially underestimating the true effects of ETI on body composition. While bone density data were available for all participants at the primary time points (pre‐ETI and postDXA1), only 69% had the longer‐term postDXA2 scan, which may introduce bias if participants with declining health were less likely to undergo follow‐up scanning. The incomplete datasets increase the risk of Type 2 error, potentially failing to detect true changes in body composition or bone density with prolonged ETI treatment. Additionally, 66% of participants were pretreated with other CFTR modulators, which may have influenced baseline bone and body composition parameters. The retrospective observational design limits our ability to establish causality. Finally, nearly all participants had at least one F508del mutation, which may limit generalizability to other CF genotypes.

## 5 Conclusions

This real‐world analysis of DXA scans in adults with CF demonstrates important changes in bone density and body composition following ETI initiation. The study reveals a biphasic pattern of bone density changes: significant decline occurred during the first 1.5 years of ETI treatment at all measured sites (lumbar spine, left hip, and right hip), but this decline was attenuated in participants with a longer follow‐up period at 4 years, suggesting potential stabilization of bone health with prolonged ETI therapy. Moreover, body composition analysis showed increases in body weight and fat mass without corresponding increases in lean mass. These findings have important implications for the long‐term management of adults with CF, as longer life expectancy with highly effective CFTR modulators necessitates attention to age‐related complications including bone health. Larger and longer studies will be needed to confirm this pattern and understand the long‐term effects of ETI on parameters that will play important roles in the health of aging pwCF.

## Author Contributions

Conceptualization: Susanne Ursula Trost, Anvitha Ankireddypalli, Tasma Harindhanavudhi, Amir Moheet. Writing: Susanne Ursula Trost, Tasma Harindhanavudhi, Amir Moheet. Data curation: Susanne Ursula Trost, Tasma Harindhanavudhi, Anvitha Ankireddypalli, Azmi Simrah, Sreekant Avula, Amir Moheet. Statistics: Qi Wang.

## Conflicts of Interest

The authors declare no conflicts of interest.

## Supporting information


**Supporting Table 1:** DXA scan results of bone mineral density in pwCF before and after ETI initiation in 43 subjects with data at all 4 time points.

## Data Availability

The data sets used during the current study are available from the corresponding author upon reasonable request.
