# Supplementary material for: Elexacaftor/Tezacaftor/Ivacaftor Effect on Bone Density and Body Composition: A Retrospective Analysis
Source: Pediatr Pulmonol. 2025 Sep 9;60(9):e71280. doi: 10.1002/ppul.71280 (PMC12418912; doi:10.1002/ppul.71280)
Supplement: Supplementary file 1 — Supporting Table 1: DXA scan results of bone mineral density in pwCF before and after ETI initiation in 43 subjects with data at all 4 time points. [file PPUL-60-0-s001.docx]

**Supplemental Table 1**

DXA scan results of bone mineral density in pwCF before and after ETI initiation in 43 subjects with data at all 4 time points.

| **Bone Mineral Density** | | | | | | **Pairwise comparison p-value** | | |
| --- | --- | --- | --- | --- | --- | --- | --- | --- |
|  | **Remote**  (n=43) | **Pre**  (n=43) | **Post1**  (n=43) | **Post2**  (n=43) | **Overall p-value** | **Remote vs Pre** | **Pre vs Post1** | **Post1 vs Post2** |
| Z-score  Lumbar spine | -0.10 (1.20) | -0.17 (1.12) | -0.39 (1.24) | -0.43 (1.16) | <0.01 | 0.87 | 0.05 | 0.94 |
| Z-score  left hip | 0.08 (1.03) | -0.03 (1.02) | -0.22 (0.99) | -0.24 (0.99) | <0.01 | 0.14 | 0.03 | 0.54 |
| Z-score  right hip | 0.10 (1.08) | -0.05 (1.01) | -0.21 (1.04) | -0.23 (1.00) | <0.01 | 0.04 | 0.03 | 0.98 |

DXA: Dual energy X-ray absorptiometry

pwCF: people with cystic fibrosis

ETI: Elexacaftor/tezacaftor/ivacaftor

Remote: the DXA scan preceding the most recent DXA done prior to ETI start

Pre: the most recent DXA prior to ETI start

Post1: the DXA scan following the initiation of ETI

Post2: the second DXA scan after the initiation of ETI
